# Anti-Inflammatory Diet and Dementia in Older Adults With Cardiometabolic Diseases

**DOI:** 10.1001/jamanetworkopen.2024.27125

**Published:** 2024-08-12

**Authors:** Abigail Dove, Michelle M. Dunk, Jiao Wang, Jie Guo, Rachel A. Whitmer, Weili Xu

**Affiliations:** 1 Aging Research Center, Department of Neurobiology, Care Sciences and Society, Karolinska Institutet, Stockholm, Sweden; 2 Department of Epidemiology and Biostatistics, School of Public Health, Tianjin Medical University, Tianjin, China; 3 Department of Epidemiology, College of Preventive Medicine, Army Medical University, Chongqing, China; 4 Department of Nutrition and Health, China Agricultural University, Beijing, China; 5 Department of Public Health Sciences and Neurology, University of California, Davis

## Abstract

**Importance:**

Inflammation has been proposed as a mechanism linking cardiometabolic diseases (CMDs) to increased risk of dementia. However, whether an anti-inflammatory diet can support brain and cognitive health among people with CMDs is unclear.

**Objective:**

To examine CMD status and dietary inflammatory potential in association with dementia risk and brain magnetic resonance imaging (MRI) measures using joint effect analysis.

**Design, Setting, and Participants:**

The UK Biobank is an ongoing community-based cohort study with baseline assessments conducted between March 13, 2006, and October 1, 2010. The present study included 84 342 dementia-free older adults (≥60 years), who were followed up until January 20, 2022 (maximum, 15 years). A subsample (n = 8917) underwent brain MRI scans between May 2, 2014, and March 13, 2020.

**Exposures:**

Baseline CMDs (including type 2 diabetes, heart disease, and stroke) were ascertained from medical records. Dietary Inflammatory Index scores (anti-inflammatory [≤−1.5 points], neutral [>−1.5 to <0.5 points], or proinflammatory [≥0.5 points]) were calculated from participants’ average intake of 31 nutrients, assessed up to 5 times using the Oxford WebQ, a web-based, 24-hour dietary assessment.

**Main Outcomes and Measures:**

Incident dementia was identified through linkage to medical records. Regional brain volumes were collected from brain MRI scans.

**Results:**

The study included 84 342 participants (mean [SD] age, 64.1 [2.9] years; 43 220 [51.2%] female). At baseline, 14 079 (16.7%) had at least 1 CMD. Over a median follow-up of 12.4 (IQR, 11.8-13.1) years, 1559 individuals (1.9%) developed dementia. With the use of joint effect analysis, the hazard ratio of dementia was 2.38 (95% CI, 1.93-2.93) for people with CMDs and a proinflammatory diet and 1.65 (95% CI, 1.36-2.00) for those with CMDs and an anti-inflammatory diet (reference: CMD-free, anti-inflammatory diet). Dementia risk was 31% lower (hazard ratio, 0.69; 95% CI, 0.55-0.88; *P* = .003) among people with CMDs and an anti-inflammatory diet. On brain MRI, participants with CMDs and an anti-inflammatory diet compared with a proinflammatory diet additionally had significantly larger gray matter volume (β = −0.15; 95% CI, −0.24 to −0.06 vs β = −0.27; 95% CI, −0.38 to −0.16) and smaller white matter hyperintensity volume (β = 0.05; 95% CI, −0.04 to 0.14 vs β = 0.16; 95% CI, 0.05-0.27).

**Conclusions and Relevance:**

In this cohort study, people with CMDs and an anti-inflammatory compared with proinflammatory diet had a significantly lower hazard ratio of dementia, larger gray matter volume, and smaller white matter hyperintensity volume.

## Key Points

**Question:**

Can an anti-inflammatory diet support brain and cognitive health among people with cardiometabolic diseases (CMDs)?

**Findings:**

In this cohort study including 84 342 older adults from the UK Biobank, participants with CMDs and an anti-inflammatory diet compared with a proinflammatory diet had a 31% lower risk of dementia. In addition, significantly larger gray matter volume and significantly lower burden of white matter hyperintensities were observed on brain magnetic resonance imaging in those with the anti-inflammatory diet.

**Meaning:**

The findings of this study suggest that lower dietary inflammatory potential may support brain and cognitive health among people with CMDs.

## Teaser

This cohort study examines the risk of dementia in individuals with cardiometabolic diseases following anti-inflammatory vs proinflammatory diets.

## Introduction

Cardiometabolic diseases (CMDs), such as type 2 diabetes,^1,2^ heart disease,^3^ and stroke,^4^ have been linked to increased risk of dementia, especially when they co-occur.^5,6,7,8^ Inflammation is implicated in the pathophysiologic characteristics of both CMDs^9^ and dementia,^10,11^ and offers a potential mechanistic explanation for the increased risk of dementia among people with CMDs.

Dietary intake can influence systemic inflammatory processes in the body.^12^ For example, the Western dietary pattern—characterized by red meat, high-fat dairy, eggs, refined grains, and processed foods—has been associated with higher levels of inflammatory biomarkers, such as C-reactive protein, interleukin 6, and tumor necrosis factor α.^12^ On the contrary, dietary patterns characterized by consumption of higher amounts of vegetables, fruits, whole grains, fish, and legumes tend to be associated with lower levels of these biomarkers.^12^

The Dietary Inflammatory Index (DII) was developed to capture the inflammatory potential of an individual’s diet based on consumption of various macronutrients and micronutrients, bioactive components, foods, and spices.^13^ In addition to reduced risk of diabetes, cardiovascular disease, and other cardiometabolic risk factors,^14,15,16^ lower dietary inflammatory potential, as measured by the DII, has been linked to lower dementia risk,^17,18,19^ better cognitive function,^18,20,21,22^ and more favorable magnetic resonance imaging (MRI) markers of brain aging.^19,23^ However, the extent to which an anti-inflammatory diet may support brain and cognitive health among people with CMDs has not yet been explored. Using 15-year longitudinal data from more than 80 000 older adults in the UK Biobank, including more than 8000 who underwent brain MRI, we examined the role of CMDs and dietary inflammatory potential in dementia risk and MRI markers of neurodegenerative and vascular brain damage using joint effect analysis.

## Methods

### Study Design and Population

The UK Biobank is an ongoing longitudinal study including more than 500 000 adults between the ages of 40 and 70 years from across the UK.^24^ Between March 13, 2006, and October 1, 2010, participants underwent a baseline examination consisting of physical and medical assessments and a series of questionnaires on sociodemographic and lifestyle factors. Between May 2, 2014, and March 13, 2020, a subset of participants additionally underwent a brain MRI scan. Changes in health status were monitored via linkage with medical records for a maximum of 15 years (until January 20, 2022). Data collection procedures were approved by the UK National Research Ethics Service and all participants provided informed consent; no financial compensation was provided. This study followed the Strengthening the Reporting of Observational Studies in Epidemiology (STROBE) reporting guideline.

Selection of the study population is illustrated in eFigure 1 in Supplement 1. The analysis was restricted to participants aged 60 years or older at baseline who completed a dietary questionnaire (n = 84 559). We further excluded 39 individuals with prevalent dementia, 53 with type 1 diabetes, and 147 with missing information on baseline CMD status, leaving a sample of 84 342 for the main analyses. The neuroimaging subsample consisted of 8917 participants who underwent a brain MRI scan over the follow-up period and were free from chronic neurologic diseases (eTable 1 in Supplement 1) at the time of the MRI scan.

### Assessment of CMDs

Cardiometabolic diseases included type 2 diabetes, heart disease, and stroke^25,26,27^ (eTable 2 in Supplement 1). Type 2 diabetes was diagnosed based on medical records, use of glucose-lowering medications, biochemical measures (hemoglobin A_1c_ ≥6.5% [to convert to proportion of total hemoglobin, multiply by 0.01]), and self-reported history of diabetes. Heart disease (including myocardial infarction, atrial fibrillation, and heart failure) and stroke were ascertained based on medical records and self-reported medical history. Cardiometabolic disease status was defined according to participants’ total number of CMDs (0, 1, or ≥2) and dichotomized as CMD-free vs CMDs.

### Dietary Assessment

Dietary data were collected using the Oxford WebQ, a web-based 24-hour dietary assessment administered at baseline and up to 4 additional times via email between February 8, 2011, and June 15, 2012.^28^ The assessment measured intake of 206 foods and 32 drinks. Energy and nutrient intake were calculated by multiplying the consumption frequency of each food and drink by a standard portion size and the nutrient composition of that item.^29^ To minimize the impact of potential inaccuracies in dietary recall, data were averaged from all available dietary assessments.^29^

### Dietary Inflammatory Index

The DII is a literature-derived population-based measure of dietary inflammatory potential.^13,30^ Briefly, the DII is based on 45 inflammation-related dietary parameters (eg, macronutrients and micronutrients, bioactive components, foods, and spices). Each dietary parameter has been assigned an inflammatory effect score (IES) (negative for anti-inflammatory and positive for proinflammatory) based on meta-analysis of 1943 previous studies measuring the association of these dietary parameters with biomarkers of inflammation and an estimate of global mean consumption based on 11 food consumption datasets (eTable 3 in Supplement 1). The DII score is calculated as the sum of each dietary parameter’s IES multiplied by the participant’s central percentile of consumption ( ∑ *^n^_i_* _ = 1_ IES*_i_* consumption_i_) using as many of the 45 dietary parameters for which information is available.

Our DII calculation was based on 31 dietary parameters (eTable 3 in Supplement 1), in line with previous studies which typically include 25 to 30.^30^ In our sample, DII ranged from −6.7 to 5.2 points and was considered as both a continuous variable and a categorical variable, tertiled as anti-inflammatory (≤−1.5 points), neutral (>−1.5 to <0.5 points), or proinflammatory (≥0.5 points).

### Dementia Diagnosis

Dementia was diagnosed based on information from inpatient records, self-reported medical history, and death registers, which was algorithmically combined to identify dementia.^31^ In validation studies, this method for dementia diagnosis has shown a positive predictive value of 82.5%.^32^

### Brain MRI Acquisition and Preprocessing

Detailed descriptions of the UK Biobank brain MRI image acquisition and processing protocols have been previously published.^33,34,35^ Briefly, T1 and T2 fluid-attenuated inversion recovery imaging was performed (Siemens Skyra 3T scanner) (eTable 4 in Supplement 1). We examined total brain volume (TBV), gray matter volume (GMV), white matter volume (WMV), hippocampal volume (HV), and white matter hyperintensity volume (WMHV), all of which were normalized for intracranial volume. To enable comparison, values for TBV, GMV, WMV, and HV were converted to *z* scores. Values for WMHV were instead log-transformed, given their skewed distribution.

### Covariates

Educational level (college/university vs not) was dichotomized based on the highest level of formal education attained. Socioeconomic status was assessed using the Townsend Deprivation Index, a measure of neighborhood-level socioeconomic deprivation.^36^ Race and ethnicity, factors that could differentially influence the development both CMDs and dementia, were self-reported according to the 2001 UK census categories and dichotomized as White compared with other race and ethnicity (including Asian, Black, multiracial, or other). Height and weight were measured during the baseline examination and used to calculate body mass index.^37^ Hypertension was defined based on blood pressure measurement (systolic ≥140 mm Hg, diastolic ≥90 mm Hg), antihypertensive medication use, medical records, and/or self-reported history of high blood pressure. Smoking status was self-reported as never, previous, or current. Physical activity was classified as inactive, moderate, or active based on the International Physical Activity Questionnaire.^38^ Apolipoprotein E (*APOE*) was dichotomized as carrier vs noncarrier of the ε4 allele.

### Statistical Analysis

Baseline characteristics of the study participants by CMD status were assessed using χ^2^ tests for categorical variables and *t* tests for continuous variables. Cox proportional hazards regression models were used to estimate the hazard ratios (HRs) and 95% CIs for the associations of CMDs and diet category with dementia. Age was used as the timescale and defined as age at baseline until age at dementia diagnosis, death, or the last available follow-up (January 20, 2022), whichever came first. The proportional hazard assumption was tested using Schoenfeld residuals; no violations were observed. We additionally modeled the HR of dementia in relation to continuous DII score using restricted cubic splines with 3 knots at fixed percentiles (10th, 50th, and 90th) of the DII distribution. Next, using joint effect analysis, we incorporated into the Cox proportional hazards regression model a 6-category indicator variable that combined CMD status (yes vs no) and diet category (anti-inflammatory, neutral, or proinflammatory) (reference: CMD-free/anti-inflammatory diet). The difference in dementia risk between the CMD/anti-inflammatory diet and CMD/proinflammatory diet groups was statistically tested by repeating the models using the CMD/proinflammatory group as the reference. Laplace regression was used to estimate the percentile differences in time (years) to dementia onset as a function of joint CMD and dietary status. In addition, linear regression models were used to estimate β coefficients and 95% CIs for the association of joint CMD and dietary status with TBV, GMV, WMV, HV, and WMHV in the neuroimaging subsample.

Multiplicative interactions between CMD status and dietary category were assessed by incorporating the CMD status × diet category cross-product term into the models. Additive interactions were assessed using relative excess risk due to interaction.

Models were first adjusted for baseline age, sex, educational level, and energy intake, followed by socioeconomic status, race and ethnicity, vascular risk factors (ie, body mass index, hypertension, smoking, and physical activity), and *APOE* ε4 carrier status. For brain MRI outcomes, we additionally adjusted for time between baseline and MRI scan, assessment center, and head and table position within the MRI scanner. Missing values for covariates were imputed using fully conditional specification, with estimates pooled from 5 iterations.

In sensitivity analyses, we (1) repeated the analysis using nonimputed data, (2) excluded individuals who received a dementia diagnosis within the first 5 years of follow-up (n = 109) (to minimize the influence of possible cases of prodromal or undiagnosed dementia at baseline), (3) accounted for the competing risk of death using Fine and Gray regression, (4) restricted the sample to participants who completed 2 or more dietary assessments (n = 51 182) to account for potential inaccuracies in 24-hour dietary recall, and (5) accounted for potential changes in dietary pattern over time by repeating the analyses using information from only the baseline dietary assessment (n = 29 175). We additionally tested the association between DII scores and systemic inflammation (ie, C-reactive protein level measured from blood samples collected at baseline) and evaluated the stability of DII scores across all dietary assessments.

All analyses were performed using Stata SE, version 16.0 (StataCorp LLC). With 2-tailed testing, *P* < .05 was considered statistically significant.

## Results

### Baseline Characteristics

At baseline, the mean (SD) age of the 84 342 participants was 64.1 (2.9) years; 43 220 (51.2%) were female, 41 122 (48.8%) were male, and 30 876 (36.8%) were college or university educated. A total of 14 079 (16.7%) participants had at least 1 CMD at study entry; these individuals were more likely to be older, be male, and identify as Asian, Black, multiracial, or another race and ethnicity; have lower educational attainment and socioeconomic status; have a higher body mass index; smoke; be physically inactive; and have hypertension (Table 1). Compared with the main analytical sample, the neuroimaging subsample (n = 8917) was younger with higher socioeconomic status and a more favorable vascular risk factor profile (eTable 5 and eTable 6 in Supplement 1).

**Table 1.** Baseline Characteristics of the Study Sample (N = 84 342)^a^

Characteristic	Full sample (N = 84 342)	By CMD status		
		CMD-free (n = 70 263)	CMD (n = 14 079)	*P* value
Age, mean (SD), y	63.9 (2.8)	63.7 (2.7)	64.4 (2.8)	<.001
Sex				
Female	43 220 (51.2)	38 375 (54.6)	4845 (34.3)	<.001
Male	41 122 (48.8)	31 888 (45.4)	9234 (65.6)	
College/university educated	30 876 (36.8)	26 538 (38.0)	4338 (31.1)	<.001
White race^b^	78 587 (93.5)	65 576 (93.7)	13 011 (92.8)	<.001
Townsend Deprivation Index, mean (SD)	−1.9 (2.7)	−1.9 (2.7)	−1.5 (2.9)	<.001
BMI, mean (SD)	27.1 (4.4)	26.7 (4.1)	29.1 (4.9)	<.001
Underweight (<20)	1685 (2.0)	1558 (2.2)	127 (1.0)	<.001
Normal weight (20 to <25)	26 757 (31.8)	24 187 (34.5)	2570 (18.3)	
Overweight (25 to <30)	37 634 (44.8)	31 433 (44.9)	6201 (44.2)	
Obese (≥30)	18 027 (21.4)	12 901 (18.4)	5126 (36.6)	
Smoking				
Never	43 157 (51.3)	37 211 (53.1)	5946 (42.4)	<.001
Former	35 984 (42.8)	28 887 (41.2)	7097 (50.7)	
Current	4960 (5.9)	3991 (5.7)	969 (6.9)	
Physical activity				
Low	11 698 (16.7)	9206 (15.8)	2492 (21.3)	<.001
Moderate	30 235 (43.2)	25 249 (43.3)	4986 (42.7)	
High	28 080 (40.1)	23 883 (40.9)	4197 (36.0)	
Hypertension	29 611 (35.2)	20 981 (29.9)	8630 (61.8)	<.001
*APOE* ε4 carrier	19 316 (27.7)	16 120 (27.6)	3196 (28.0)	.35
Energy intake, mean (SD), kcal	2035 (518)	2033 (514)	2045 (540)	.02
DII score, mean (SD)	−0.6 (2.0)	−0.7 (2.0)	−0.6 (2.0)	<.001
Proinflammatory (≥0.5 pts)	29 901 (35.5)	20 122 (28.6)	4295 (30.5)	<.001
Neutral (>−1.5 to <0.5 pts)	30 024 (35.6)	25 200 (35.9)	4824 (34.3)	
Anti-inflammatory (≤−1.5 pts)	24 417 (29.0)	24 941 (35.5)	4960 (35.2)	

Abbreviations: *APOE*, apolipoprotein E; BMI, body mass index (calculated as weight in kilograms divided by height in meters squared); CMD, cardiometabolic diseases; DII, Dietary Inflammatory Index.

^a^ Missing participant data: 515 for educational level; 330 for race and ethnicity; 76 for Townsend Deprivation Index; 239 for BMI; 241 for smoking status; 14 329 for physical activity level; 121 for hypertension; and 14 566 for *APOE* ε4 status.

^b^ Race and ethnicity was self-reported according to the 2001 UK census categories. The other group included Asian, Black, multiracial, or other (ie, any other race or ethnicity not already specified).

### CMDs, Dietary Inflammatory Potential, and Dementia

**Figure 1.**
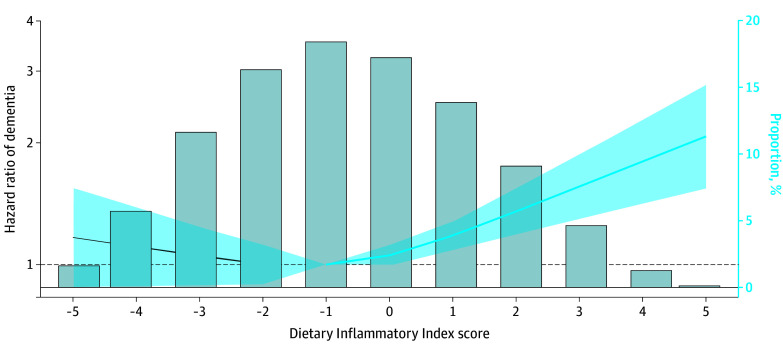
Association Between Dietary Inflammatory Potential and Dementia Risk Gray bars represent the distribution of Dietary Inflammatory Index score in the study population. The association between the DII score and dementia risk is modeled using restricted cubic splines adjusted for age, sex, educational level, energy intake, race and ethnicity, socioeconomic status, body mass index, smoking status, physical activity, hypertension, apolipoprotein E ε4 carrier status, and cardiometabolic disease status. The blue line and blue shaded areas represent the hazard ratio and 95% CI of dementia as a function of the DII score. The reference level is set as the median DII score in the population (−1 pts).

Over the follow-up period (median, 12.4 [IQR, 11.8-13.1] years), a total of 1559 participants (1.9%) developed dementia. The presence of CMDs was associated with an 81% increased risk of dementia (HR, 1.81; 95% CI, 1.61-2.04]) (Table 2). However, compared with the proinflammatory diet, having an anti-inflammatory diet was associated with 21% lower dementia risk (HR, 0.79; 95% CI, 0.68-0.91). Consistent with this, when considered as a continuous variable, a higher DII score was associated with a significantly increased risk of dementia (Figure 1).

**Table 2.** Association of CMD Status and Dietary Inflammatory Potential Level With Dementia Risk: Results From Cox Proportional Hazards Regression Models

CMD and dietary inflammatory potential status	Participants, No.	Hazard ratio of dementia (95% CI)^a^	
		Basic adjusted	Multiadjusted
CMD status			
CMD-free	70 263	1 [Reference]	1 [Reference]
CMDs	14 079	1.86 (1.67-2.09)	1.81 (1.61-2.04)
1	12 236	1.70 (1.51-1.92)	1.67 (1.47-1.89)
≥2	1843	3.00 (2.44-3.70)	2.87 (2.31-3.56)
Dietary inflammatory potential			
Proinflammatory (DII ≥0.5 pts)	24 417	1 [Reference]	1 [Reference]
Neutral (DII >−1.5 to <0.5 pts)	30 024	0.84 (0.73-0.95)	0.84 (0.74-0.96)
Anti-inflammatory (DII ≤−1.5 pts)	29 901	0.80 (0.69-0.92)	0.79 (0.68-0.91)
Joint exposure			
CMDs, diet			
CMD-free			
Anti-inflammatory	24 941	1 [Reference]	1 [Reference]
Neutral	25 200	1.02 (0.88-1.18)	1.04 (0.89-1.20)
Proinflammatory	20 122	1.20 (1.02-1.41)	1.21 (1.02-1.42)
CMDs			
Anti-inflammatory	4960	1.69 (1.40-2.04)	1.65 (1.36-2.00)
Neutral	4824	1.96 (1.62-2.37)	1.91 (1.57-2.32)
Proinflammatory	4295	2.41 (1.96-2.95)	2.38 (1.93-2.93)

Abbreviations: CMD, cardiometabolic diseases; DII, Dietary Inflammatory Index.

^a^ Basic-adjusted models included age at baseline, sex, educational level, and energy intake. Multiadjusted models additionally included race and ethnicity, socioeconomic status, body mass index, smoking status, physical activity, hypertension, apolipoprotein E ε4 carrier status, and CMD status or dietary inflammatory potential category as appropriate.

### Joint Effect Analysis of CMDs and Dietary Inflammatory Potential in Dementia Risk

**Figure 2.**
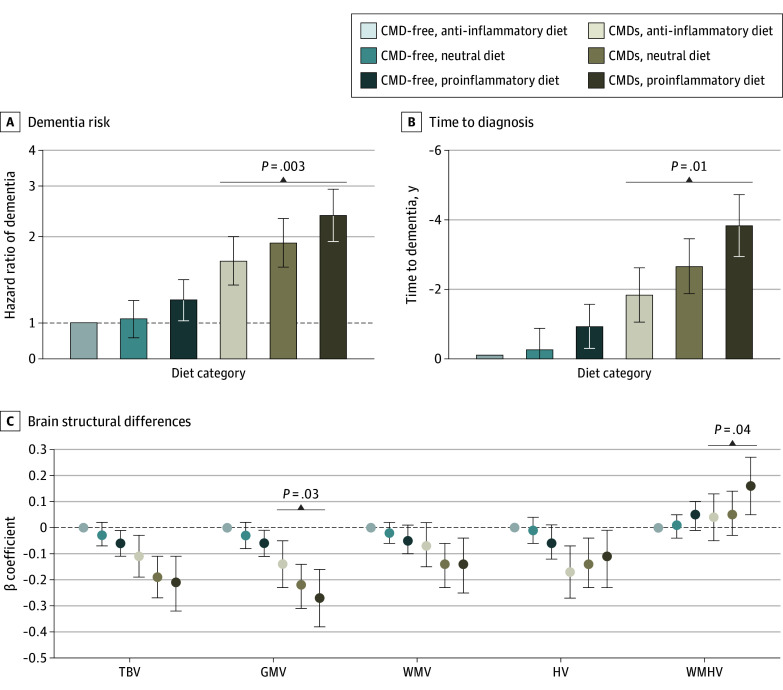
Joint Effect Analysis of Cardiometabolic Disease (CMD) Status and Dietary Inflammatory Potential in Dementia Risk, Time to Dementia Diagnosis, and Brain Structural Differences Cox proportional hazards regression models for dementia risk (A) and Laplace regression models for time to dementia diagnosis (B) were adjusted for age, sex, educational level, energy intake, race and ethnicity, socioeconomic status, body mass index, smoking status, physical activity, hypertension, and apolipoprotein E ε4 carrier status. Linear regression models for brain structural differences (C) were additionally adjusted for time between baseline and magnetic resonance imaging scan, assessment center, and head and table position within the scanner. In all models, significant differences between the CMD with anti-inflammatory diet and CMD with proinflammatory diet groups were assessed by repeating the models using the CMD with proinflammatory diet group as the reference. GMV indicates gray matter volume; HV, hippocampal volume; TBV, total brain volume; WMHV, white matter hyperintensity volume; and WMV, white matter volume.

In joint effect analysis, the HR of dementia was 2.38 (95% CI, 1.93-2.93) for individuals with CMDs and a proinflammatory diet, 1.91 (95% CI, 1.57-2.32) for those with CMDs and a neutral diet, and 1.65 (95% CI, 1.36-2.00) for those with CMDs and an anti-inflammatory diet (reference: CMD-free, anti-inflammatory diet) (Table 2). Dementia risk was 31% lower (HR, 0.69; 95% CI, 0.55-0.88; *P* = .003) among people with CMDs and an anti-inflammatory diet as opposed to proinflammatory diet (Figure 2A). Significant multiplicative (*P* < .001) and additive (relative excess risk due to interaction = 0.48; 95% CI, 0.01-0.97; *P* = .04) interactions were detected between CMD status and diet category on dementia risk. In Laplace regression, participants with CMDs and an anti-inflammatory diet developed dementia 2 years later than those with CMDs and a proinflammatory diet (Figure 2B).

### CMDs, Dietary Inflammatory Potential, and Brain MRI

The presence of CMDs was associated with significantly smaller TBV, GMV, WMV, and HV, and significantly larger WMHV. In contrast, compared with a proinflammatory diet, an anti-inflammatory diet was associated with larger TBV, GMV, and WMV, smaller WMHV, but no significant difference in HV (Table 3).

**Table 3.** Association of CMD Status and Dietary Inflammatory Potential Level With Neuroimaging Measures: Results From Linear Regression Models^a^

CMD and dietary inflammatory potential status	Participants, No.	β (95% CI)				
		Total brain volume	Gray matter volume	White matter volume	Hippocampal volume	White matter hyperintensity volume
CMD status						
CMD-free	7773	1 [Reference]	1 [Reference]	1 [Reference]	1 [Reference]	1 [Reference]
CMDs	1144	−0.14 (−0.20 to −0.09)	−0.18 (−0.24 to −0.13)	−0.10 (−0.15 to −0.04)	−0.13 (−0.19 to −0.07)	0.07 (0.01 to 0.12)
Dietary inflammatory potential						
Proinflammatory (DII ≥0.5 pts)	2235	1 [Reference]	1 [Reference]	1 [Reference]	1 [Reference]	1 [Reference]
Neutral (DII >−1.5 to <0.5 pts)	3315	0.03 (−0.02 to 0.07)	0.03 (−0.02 to 0.07)	0.03 (−0.02 to 0.07)	0.04 (−0.02 to 0.09)	−0.05 (−0.10 to 0.01)
Anti-inflammatory (DII ≤−1.5 pts)	3367	0.06 (0.01 to 0.11)	0.06 (0.01 to 0.12)	0.05 (0.01 to 0.10)	0.04 (−0.02 to 0.10)	−0.05 (−0.10 to −0.01)
Joint exposure						
CMDs, diet						
CMD-free						
Anti-inflammatory	2951	1 [Reference]	1 [Reference]	1 [Reference]	1 [Reference]	1 [Reference]
Neutral	2863	−0.03 (−0.07 to 0.02)	−0.03 (−0.08 to 0.01)	−0.02 (−0.06 to 0.02)	−0.01 (−0.06 to 0.04)	0.01 (−0.04 to 0.05)
Proinflammatory	1959	−0.06 (−0.11 to −0.01)	−0.06 (−0.11 to −0.01)	−0.05 (−0.10 to 0.01)	−0.05 (−0.12 to 0.01)	0.04 (−0.01 to 0.10)
CMDs						
Anti-inflammatory	416	−0.11 (−0.20 to −0.03)	−0.15 (−0.24 to −0.06)	−0.07 (−0.15 to 0.01)	−0.18 (−0.28 to −0.08)	0.05 (−0.04 to 0.14)
Neutral	452	−0.19 (−0.27 to −0.11)	−0.22 (−0.31 to −0.14)	−0.14 (−0.23 to −0.06)	−0.14 (−0.23 to −0.04)	0.05 (−0.04 to 0.14)
Proinflammatory	276	−0.21 (−0.32 to −0.11)	−0.27 (−0.38 to −0.16)	−0.14 (−0.25 to −0.04)	−0.11 (−0.23 to 0.01)	0.16 (0.05 to 0.27)

Abbreviations: CMD, cardiometabolic diseases; DII, Dietary Inflammatory Index.

^a^ All models were adjusted for age, sex, educational level, energy intake, race and ethnicity, socioeconomic status, body mass index, smoking status, physical activity, hypertension, apolipoprotein E ε4 carrier status, magnetic resonance imaging (MRI)–related factors (time between baseline and MRI scan, assessment center, and head and table position within the scanner), and CMD status or dietary inflammatory potential category as appropriate.

With our use of joint effect analysis, participants with CMDs and an anti-inflammatory compared with a proinflammatory diet had significantly larger GMV (β = −0.15; 95% CI, −0.24 to −0.06 vs β = −0.27; 95% CI, −0.38 to −0.16) and significantly smaller WMHV (β = 0.05; 95% CI, −0.04 to 0.14 vs β = 0.16; 95% CI, 0.05-0.27) (Figure 2C). Multiplicative interactions were detected between CMD status and diet category for TBV (*P* < .001), GMV (*P* < .001), WMV (*P* = .007), and WMHV (*P* = .005), but not HV (*P* = .09).

### Additional Analyses

In sensitivity analysis, similar results were obtained when we repeated the analyses using nonimputed data (eTable 7 in Supplement 1) and after excluding 109 individuals who received a dementia diagnosis within the first 5 years of follow-up (eTable 8 in Supplement 1). The HRs of all-cause dementia were slightly attenuated in Fine and Gray regression models (eTable 9 in Supplement 1), indicating that the results may have been affected by the competing risk of death. We confirmed that higher DII scores were associated with higher levels of systemic inflammation (eTable 10 in Supplement 1) and that DII scores remained stable across the multiple dietary assessments (eTable 11 and eFigure 2 in Supplement 1). Accordingly, results remained consistent in analyses restricted to participants who underwent multiple dietary assessments (eTable 12 in Supplement 1) or using data from only the baseline dietary assessment (eTable 13 in Supplement 1).

## Discussion

In this large-scale study, we found that, among people with CMDs, following an anti-inflammatory diet compared with a proinflammatory diet was associated with a lower risk of dementia and significantly lower levels of MRI markers of neurodegenerative and vascular brain damage. The association between CMDs and increased risk of dementia has been emphasized in recent studies,^6,7,8^ but few strategies for reducing dementia risk among older adults with CMDs have been identified. The present study highlights an anti-inflammatory diet as one potential approach. Over 15 years of follow-up, dementia risk was 31% lower in participants with CMDs and an anti-inflammatory diet compared with a proinflammatory diet. We further found that people with CMDs and an anti-inflammatory diet developed dementia 2 years later than those with a proinflammatory diet.

Higher DII scores (ie, a proinflammatory diet) have been associated with a significantly increased risk of dementia in previous longitudinal studies, including the Hellenic Longitudinal Investigation of Aging and Diet (n = 1059),^17^ the Women’s Health Initiative Memory Study (n = 7085),^18^ and the UK Biobank (n = 166 377).^19^ Moreover, several studies have reported an inverse association between DII score and cognitive function, specifically global cognition,^18,20,21,39^ executive function,^22^ and episodic,^22^ semantic,^22^ and verbal^20^ memory. The present study adds to this literature by observing that an anti-inflammatory diet may attenuate the HR of dementia among people with CMDs.

To complement these findings, we explored the association of CMD status and diet with brain MRI phenotypes using joint effect analysis. Individuals with CMDs and an anti-inflammatory diet compared with a proinflammatory diet had significantly higher GMV (indicating less neurodegeneration) and significantly lower WMHV (indicating less vascular injury). This is consistent with previous investigations linking higher DII scores to smaller GMV,^19,23^ lower lateral ventricular volume,^23^ and larger WMHV,^19^ although at least one study reported no association between DII score and brain MRI markers.^40^ Longitudinal brain MRI studies are needed to better understand the interaction between dietary inflammatory potential and brain disease, especially in the context of other risk factors like CMDs.

Our results can be interpreted within the framework of inflammaging, the theory that aging and the development of diseases in older persons is due to a breakdown in the normal balance of proinflammatory and anti-inflammatory processes as individuals age.^41^ Acute activation of inflammatory processes can be adaptive in response to pathogens and tissue damage, but chronic, low-grade inflammation has been linked to an increased risk of several age-related disorders, including cardiovascular disease,^42^ type 2 diabetes,^43^ and dementia.^10,11^ A potential explanation for our results is that an anti-inflammatory diet might dampen systemic inflammation (especially among people with CMDs), thereby slowing the development of dementia. Moreover, the presence of significant interactions between CMD status and dietary inflammatory potential in the present study could suggest that the potential benefits of an anti-inflammatory diet for cognitive and brain health may be more pronounced in people with CMDs. This warrants deeper investigation in future studies.

### Strengths and Limitations

Strengths of this study include the use of a community-based cohort with a large sample size and detailed data collection procedure, including brain MRI scans for more than 8000 participants. However, our findings should be considered in the context of several limitations. First, healthy volunteer bias in the UK Biobank,^44,45^ particularly in the neuroimaging subsample, could limit the generalizability of our findings and may have contributed to an underestimation of the observed associations. Second, both dementia and CMDs were ascertained primarily through medical records and therefore likely to be underdiagnosed in this study.^5,6^ Relatedly, having prodromal or as-of-yet undiagnosed dementia may contribute to the development of CMDs by making it more difficult to manage medical conditions and adhere to a healthy lifestyle. However, results remained consistent in sensitivity analyses excluding participants with likely prodromal or undiagnosed dementia (eTable 8 in Supplement 1). Additionally, dietary inflammatory potential was calculated based on self-reported dietary intake over the past 24 hours, a method that is subject to recall bias. To address this, we averaged dietary intake from up to 5 available assessments and conducted detailed sensitivity analyses to confirm the stability of DII scores over this time (eTables 11-13 in Supplement 1). Moreover, there are inherent limitations to the DII insofar as it assesses individual dietary components in isolation and therefore cannot account for the source of certain nutrients (eg, plant vs animal proteins, refined vs whole grains) or factors such as food storage and cooking methods that impact nutrient content and bioavailability.^46^ To this end, we verified the association between higher DII scores and higher levels of systemic inflammation in the study population (eTable 10 in Supplement 1), but it is possible that dietary inflammatory potential was misestimated, especially considering that all 14 of the DII components for which data were unavailable in the UK Biobank were anti-inflammatory (eTable 3 in Supplement 1). This may have contributed to an underestimation of the observed associations.^44,45^

## Conclusions

In this cohort study, participants with CMDs and an anti-inflammatory diet had a lower risk of dementia compared with those with a proinflammatory diet. Moreover, people with CMDs and an anti-inflammatory diet had significantly higher GMV and lower WMHV than their counterparts with a proinflammatory diet. Together, these results highlight an anti-inflammatory diet as a modifiable factor that may support brain and cognitive health among people with CMDs.

## Notes

Supplement 1. **eFigure 1.** Study Population Flowchart **eTable 1.** Neurological Disorders Used as Exclusion Criteria for the Neuroimaging Subsample **eTable 2.** UK Biobank Field Codes Used to Ascertain Baseline CMD Status **eTable 3.** Inflammatory Effect Scores and Globally Daily Mean Intake for 45 Dietary Parameters Included in the Dietary Inflammatory Index (DII) **eTable 4.** UK Biobank Brain MRI Image Acquisition Protocols **eTable 5.** Baseline Characteristics of the Neuroimaging Subsample (n = 8917) **eTable 6.** Comparison of Participants Included vs Not Included in the Neuroimaging Subsample **eTable 7.** Summary of Main Results Using Non-Imputed Data for Covariates **eTable 8.** Hazard Ratios (HR) From Cox Regression Models for the Associations of CMD Status and Dietary Inflammatory Potential With Dementia Risk, After Excluding 109 Study Participants Who Developed Dementia Within the First 5 Years of Follow-Up (n = 84 233) **eTable 9.** Subdistribution Hazard Ratios (SHR) From Fine & Gray Regression Models for the Associations of CMD Status and Dietary Inflammatory Potential With Dementia Risk, Accounting for the Competing Risk of Death **eTable 10.** Association Between Dietary Inflammatory Potential and CRP: Results From Linear Regression Models **eTable 11.** DII Scores Over Multiple Assessments in the Anti-Inflammatory, Neutral, and Pro-Inflammatory Diet Groups **eFigure 2.** Trajectories of DII Scores Over Multiple Assessments in the Anti-Inflammatory, Neutral, and Pro-Inflammatory Diet Groups **eTable 12.** Summary of Main Results Including Only Participants Who Completed Two or More Dietary Assessments (n = 51 182) **eTable 13.** Summary of Main Results Using DII Score Calculated Based on Only Baseline Dietary Assessment (n = 29 175)

Supplement 2.
Data Sharing Statement

## References

[1] Srikanth V, Sinclair AJ, Hill-Briggs F, Moran C, Biessels GJ. Type 2 diabetes and cognitive dysfunction—towards effective management of both comorbidities. Lancet Diabetes Endocrinol. 2020;8(6):535-545. doi:10.1016/S2213-8587(20)30118-2 32445740

[2] Biessels GJ, Strachan MWJ, Visseren FLJ, Kappelle LJ, Whitmer RA. Dementia and cognitive decline in type 2 diabetes and prediabetic stages: towards targeted interventions. Lancet Diabetes Endocrinol. 2014;2(3):246-255. doi:10.1016/S2213-8587(13)70088-3 24622755

[3] Qiu C, Fratiglioni L. A major role for cardiovascular burden in age-related cognitive decline. Nat Rev Cardiol. 2015;12(5):267-277. doi:10.1038/nrcardio.2014.223 25583619

[4] Kuźma E, Lourida I, Moore SF, Levine DA, Ukoumunne OC, Llewellyn DJ. Stroke and dementia risk: a systematic review and meta-analysis. Alzheimers Dement. 2018;14(11):1416-1426. doi:10.1016/j.jalz.2018.06.3061 30177276 PMC6231970

[5] Wang Z, Marseglia A, Shang Y, Dintica C, Patrone C, Xu W. Leisure activity and social integration mitigate the risk of dementia related to cardiometabolic diseases: a population-based longitudinal study. Alzheimers Dement. 2020;16(2):316-325. doi:10.1016/j.jalz.2019.09.003 31718906

[6] Dove A, Marseglia A, Shang Y, . Cardiometabolic multimorbidity accelerates cognitive decline and dementia progression. Alzheimers Dement. Published online June 6, 2022. doi:10.1002/alz.12708 35708183

[7] Dove A, Guo J, Marseglia A, . Cardiometabolic multimorbidity and incident dementia: the Swedish Twin Registry. Eur Heart J. 2022;00:1-11. 36577740 10.1093/eurheartj/ehac744 PMC9925275

[8] Tai XY, Veldsman M, Lyall DM, . Cardiometabolic multimorbidity, genetic risk, and dementia: a prospective cohort study. Lancet Healthy Longev. 2022;3(6):e428-e436. doi:10.1016/S2666-7568(22)00117-9 35711612 PMC9184258

[9] Donath MY, Meier DT, Böni-Schnetzler M. Inflammation in the pathophysiology and therapy of cardiometabolic disease. Endocr Rev. 2019;40(4):1080-1091. doi:10.1210/er.2019-00002 31127805 PMC6624792

[10] Darweesh SKL, Wolters FJ, Ikram MA, de Wolf F, Bos D, Hofman A. Inflammatory markers and the risk of dementia and Alzheimer’s disease: a meta-analysis. Alzheimers Dement. 2018;14(11):1450-1459. doi:10.1016/j.jalz.2018.02.014 29605221

[11] Koyama A, O’Brien J, Weuve J, Blacker D, Metti AL, Yaffe K. The role of peripheral inflammatory markers in dementia and Alzheimer’s disease: a meta-analysis. J Gerontol A Biol Sci Med Sci. 2013;68(4):433-440. doi:10.1093/gerona/gls187 22982688 PMC3693673

[12] Galland L. Diet and inflammation. Nutr Clin Pract. 2010;25(6):634-640. doi:10.1177/0884533610385703 21139128

[13] Shivappa N, Steck SE, Hurley TG, Hussey JR, Hébert JR. Designing and developing a literature-derived, population-based dietary inflammatory index. Public Health Nutr. 2014;17(8):1689-1696. doi:10.1017/S1368980013002115 23941862 PMC3925198

[14] Shivappa N, Godos J, Hébert JR, . Dietary inflammatory index and cardiovascular risk and mortality—a meta-analysis. Nutrients. 2018;10(2):1-15. doi:10.3390/nu10020200 29439509 PMC5852776

[15] Aslani Z, Sadeghi O, Heidari-Beni M, . Correction to: association of dietary inflammatory potential with cardiometabolic risk factors and diseases: a systematic review and dose-response meta-analysis of observational studies. Diabetol Metab Syndr. 2020;12(1):106. doi:10.1186/s13098-020-00615-2 33292479 PMC7712617

[16] Hariharan R, Odjidja EN, Scott D, . The dietary inflammatory index, obesity, type 2 diabetes, and cardiovascular risk factors and diseases. Obes Rev. 2022;23(1):e13349. doi:10.1111/obr.13349 34708499

[17] Charisis S, Ntanasi E, Yannakoulia M, . Diet Inflammatory Index and dementia incidence: a population-based study. Neurology. 2021;97(24):e2381-e2391. doi:10.1212/WNL.0000000000012973 34759053 PMC8673721

[18] Hayden KM, Beavers DP, Steck SE, . The association between an inflammatory diet and global cognitive function and incident dementia in older women: the Women’s Health Initiative Memory Study. Alzheimers Dement. 2017;13(11):1187-1196. doi:10.1016/j.jalz.2017.04.004 28531379 PMC5909961

[19] Shi Y, Lin F, Li Y, . Association of pro-inflammatory diet with increased risk of all-cause dementia and Alzheimer’s dementia: a prospective study of 166,377 UK Biobank participants. BMC Med. 2023;21(1):266. doi:10.1186/s12916-023-02940-5 37480061 PMC10362711

[20] Kesse-Guyot E, Assmann KE, Andreeva VA, . Long-term association between the dietary inflammatory index and cognitive functioning: findings from the SU.VI.MAX study. Eur J Nutr. 2017;56(4):1647-1655. doi:10.1007/s00394-016-1211-3 27055851

[21] Skoczek-Rubińska A, Muzsik-Kazimierska A, Chmurzynska A, Jamka M, Walkowiak J, Bajerska J. Inflammatory potential of diet is associated with biomarkers levels of inflammation and cognitive function among postmenopausal women. Nutrients. 2021;13(7):2323. doi:10.3390/nu13072323 34371834 PMC8308633

[22] Frith E, Shivappa N, Mann JR, Hébert JR, Wirth MD, Loprinzi PD. Dietary Inflammatory Index and memory function: population-based national sample of elderly Americans. Br J Nutr. 2018;119(5):552-558. doi:10.1017/S0007114517003804 29361990 PMC5839966

[23] Melo Van Lent D, Gokingco H, Short MI, . Higher Dietary Inflammatory Index scores are associated with brain MRI markers of brain aging: results from the Framingham Heart Study Offspring cohort. Alzheimers Dement. 2023;19(2):621-631. doi:10.1002/alz.12685 35522830 PMC9637238

[24] Sudlow C, Gallacher J, Allen N, . UK Biobank: an open access resource for identifying the causes of a wide range of complex diseases of middle and old age. PLoS Med. 2015;12(3):e1001779. doi:10.1371/journal.pmed.1001779 25826379 PMC4380465

[25] Di Angelantonio E, Kaptoge S, Wormser D, ; Emerging Risk Factors Collaboration. Association of cardiometabolic multimorbidity with mortality. JAMA. 2015;314(1):52-60. doi:10.1001/jama.2015.7008 26151266 PMC4664176

[26] Kivimäki M, Kuosma E, Ferrie JE, . Overweight, obesity, and risk of cardiometabolic multimorbidity: pooled analysis of individual-level data for 120813 adults from 16 cohort studies from the USA and Europe. Lancet Public Health. 2017;2(6):e277-e285. doi:10.1016/S2468-2667(17)30074-9 28626830 PMC5463032

[27] Singh-Manoux A, Fayosse A, Sabia S, . Clinical, socioeconomic, and behavioural factors at age 50 years and risk of cardiometabolic multimorbidity and mortality: a cohort study. PLoS Med. 2018;15(5):e1002571. doi:10.1371/journal.pmed.1002571 29782486 PMC5962054

[28] Liu B, Young H, Crowe FL, . Development and evaluation of the Oxford WebQ, a low-cost, web-based method for assessment of previous 24 h dietary intakes in large-scale prospective studies. Public Health Nutr. 2011;14(11):1998-2005. doi:10.1017/S1368980011000942 21729481

[29] Perez-Cornago A, Pollard Z, Young H, . Description of the updated nutrition calculation of the Oxford WebQ questionnaire and comparison with the previous version among 207,144 participants in UK Biobank. Eur J Nutr. 2021;60(7):4019-4030. doi:10.1007/s00394-021-02558-4 33956230 PMC8437868

[30] Hébert JR, Shivappa N, Wirth MD, Hussey JR, Hurley TG. Perspective: the Dietary Inflammatory Index (DII)—lessons learned, improvements made, and future directions. Adv Nutr. 2019;10(2):185-195. doi:10.1093/advances/nmy071 30615051 PMC6416047

[31] Bush K, Wilkinson T, Schnier C, Nolan J, Sudlow C. Definitions of dementia and the major diagnostic pathologies, UK Biobank phase 1 outcomes adjudication. March 2018. Accessed July 4, 2024. https://biobank.ndph.ox.ac.uk/ukb/ukb/docs/alg_outcome_dementia.pdf

[32] Wilkinson T, Schnier C, Bush K, ; Dementias Platform UK and UK Biobank. Identifying dementia outcomes in UK Biobank: a validation study of primary care, hospital admissions and mortality data. Eur J Epidemiol. 2019;34(6):557-565. doi:10.1007/s10654-019-00499-1 30806901 PMC6497624

[33] Miller KL, Alfaro-Almagro F, Bangerter NK, . Multimodal population brain imaging in the UK Biobank prospective epidemiological study. Nat Neurosci. 2016;19(11):1523-1536. doi:10.1038/nn.4393 27643430 PMC5086094

[34] Alfaro-Almagro F, Jenkinson M, Bangerter NK, . Image processing and quality control for the first 10,000 brain imaging datasets from UK Biobank. Neuroimage. 2018;166:400-424. doi:10.1016/j.neuroimage.2017.10.034 29079522 PMC5770339

[35] Smith SM, Alfaro-Almagro F, Miller KL. UK Biobank Brain Imaging Documentation. May 2024. Accessed January 20, 2023. https://biobank.ctsu.ox.ac.uk/crystal/crystal/docs/brain_mri.pdf

[36] Townsend P. Deprivation. J Soc Policy. 1987;16(2):125-146. doi:10.1017/S0047279400020341

[37] Atti AR, Palmer K, Volpato S, Winblad B, De Ronchi D, Fratiglioni L. Late-life body mass index and dementia incidence: nine-year follow-up data from the Kungsholmen Project. J Am Geriatr Soc. 2008;56(1):111-116. doi:10.1111/j.1532-5415.2007.01458.x 18028342

[38] IPAQ Group. IPAQ scoring protocol—International Physical Activity Questionnaire. Accessed January 10, 2023. https://sites.google.com/site/theipaq/scoring-protocol

[39] Shin D, Kwon SC, Kim MH, . Inflammatory potential of diet is associated with cognitive function in an older adult Korean population. Nutrition. 2018;55-56:56-62. doi:10.1016/j.nut.2018.02.026 29960158 PMC8684699

[40] Zabetian-Targhi F, Srikanth VK, Smith KJ, . Associations between the Dietary Inflammatory Index, brain volume, small vessel disease, and global cognitive function. J Acad Nutr Diet. 2021;121(5):915-924.e3. doi:10.1016/j.jand.2020.11.004 33339764

[41] Franceschi C, Capri M, Monti D, . Inflammaging and anti-inflammaging: a systemic perspective on aging and longevity emerged from studies in humans. Mech Ageing Dev. 2007;128(1):92-105. doi:10.1016/j.mad.2006.11.016 17116321

[42] Ruparelia N, Chai JT, Fisher EA, Choudhury RP. Inflammatory processes in cardiovascular disease: a route to targeted therapies. Nat Rev Cardiol. 2017;14(3):133-144. doi:10.1038/nrcardio.2016.185 27905474 PMC5525550

[43] Donath MY, Shoelson SE. Type 2 diabetes as an inflammatory disease. Nat Rev Immunol. 2011;11(2):98-107. doi:10.1038/nri2925 21233852

[44] Batty GD, Gale CR, Kivimäki M, Deary IJ, Bell S. Comparison of risk factor associations in UK Biobank against representative, general population based studies with conventional response rates: prospective cohort study and individual participant meta-analysis. BMJ. 2020;368:m131. doi:10.1136/bmj.m131 32051121 PMC7190071

[45] Fry A, Littlejohns TJ, Sudlow C, . Comparison of sociodemographic and health-related characteristics of UK Biobank participants with those of the general population. Am J Epidemiol. 2017;186(9):1026-1034. doi:10.1093/aje/kwx246 28641372 PMC5860371

[46] Lee S, Choi Y, Jeong HS, Lee J, Sung J. Effect of different cooking methods on the content of vitamins and true retention in selected vegetables. Food Sci Biotechnol. 2017;27(2):333-342. doi:10.1007/s10068-017-0281-1 30263756 PMC6049644

