# Supplementary material for: Anti-Inflammatory Diet and Dementia in Older Adults With Cardiometabolic Diseases
Source: JAMA Netw Open. 2024 Aug 12;7(8):e2427125. doi: 10.1001/jamanetworkopen.2024.27125 (PMC11320167; doi:10.1001/jamanetworkopen.2024.27125)
Supplement: Supplement 1. — eFigure 1. Study Population Flowchart eTable 1. Neurological Disorders Used as Exclusion Criteria for the Neuroimaging Subsample eTable 2. UK Biobank Field Codes Used to Ascertain Baseline CMD Status eTable 3. Inflammatory Effect Scores and Globally Daily Mean Intake for 45 Dietary Parameters Included in the Dietary Inflammatory Index (DII) eTable 4. UK Biobank Brain MRI Image Acquisition Protocols eTable 5. Baseline Characteristics of the Neuroimaging Subsample (n = 8917) eTable 6. Comparison of Participants Included vs Not Included in the Neuroimaging Subsample eTable 7. Summary of Main Results Using Non-Imputed Data for Covariates eTable 8. Hazard Ratios (HR) From Cox Regression Models for the Associations of CMD Status and Dietary Inflammatory Potential With Dementia Risk, After Excluding 109 Study Participants Who Developed Dementia Within the First 5 Years of Follow-Up (n = 84 233) eTable 9. Subdistribution Hazard Ratios (SHR) From Fine & Gray Regression Models for the Associations of CMD Status and Dietary Inflammatory Potential With Dementia Risk, Accounting for the Competing Risk of Death eTable 10. Association Between Dietary Inflammatory Potential and CRP: Results From Linear Regression Models eTable 11. DII Scores Over Multiple Assessments in the Anti-Inflammatory, Neutral, and Pro-Inflammatory Diet Groups eFigure 2. Trajectories of DII Scores Over Multiple Assessments in the Anti-Inflammatory, Neutral, and Pro-Inflammatory Diet Groups eTable 12. Summary of Main Results Including Only Participants Who Completed Two or More Dietary Assessments (n = 51 182) eTable 13. Summary of Main Results Using DII Score Calculated Based on Only Baseline Dietary Assessment (n = 29 175) [file jamanetwopen-e2427125-s001.pdf]

Supplementary Online Content

Dove A, Dunk MM, Wang J, Guo J, Whitmer RA, Xu W. Anti-inflammatory diet and dementia in older adults with cardiometabolic diseases. *JAMA Netw Open*. 2024;7(8):e2427125. doi:10.1001/jamanetworkopen.2024.2712

eFigure 1. Study Population Flowchart

eTable 1. Neurological Disorders Used as Exclusion Criteria for the Neuroimaging Subsample

eTable 2. UK Biobank Field Codes Used to Ascertain Baseline CMD Status

eTable 3. Inflammatory Effect Scores and Globally Daily Mean Intake for 45 Dietary Parameters Included in the Dietary Inflammatory Index (DII)

eTable 4. UK Biobank Brain MRI Image Acquisition Protocols

eTable 5. Baseline Characteristics of the Neuroimaging Subsample (n=8,917)

eTable 6. Comparison of Participants Included vs. Not Included in the Neuroimaging Subsample

eTable 7. Summary of Main Results Using Non-Imputed Data for Covariates

eTable 8. Hazard ratios (HR) From Cox Regression Models for the Associations of CMD Status and Dietary Inflammatory Potential With Dementia Risk, After Excluding 109 Study Participants Who Developed Dementia Within the First 5 Years of Follow-Up (n=84,233)

eTable 9. Subdistribution Hazard Ratios (SHR) From Fine & Gray Regression Models for the Associations of CMD Status and Dietary Inflammatory Potential With Dementia Risk, Accounting for the Competing Risk of Death

eTable 10. Association Between Dietary Inflammatory Potential and CRP: Results From Linear Regression Models

eTable 11. DII Scores Over Multiple Assessments in the Anti-Inflammatory, Neutral, and Pro- Inflammatory Diet Groups

eFigure 2. Trajectories of DII Scores Over Multiple Assessments in the Anti-Inflammatory, Neutral, and Pro-Inflammatory Diet Groups

eTable 12. Summary of Main Results Including Only Participants Who Completed Two or More Dietary Assessments (n=51,182)

eTable 13. Summary of Main Results Using DII Score Calculated Based on Only Baseline Dietary Assessment (n=29,175)

This supplementary material has been provided by the authors to give readers additional information about their work.

© 2024 Dove A et al. *JAMA Network Open.*

eFigure 1. Study population flowchart.

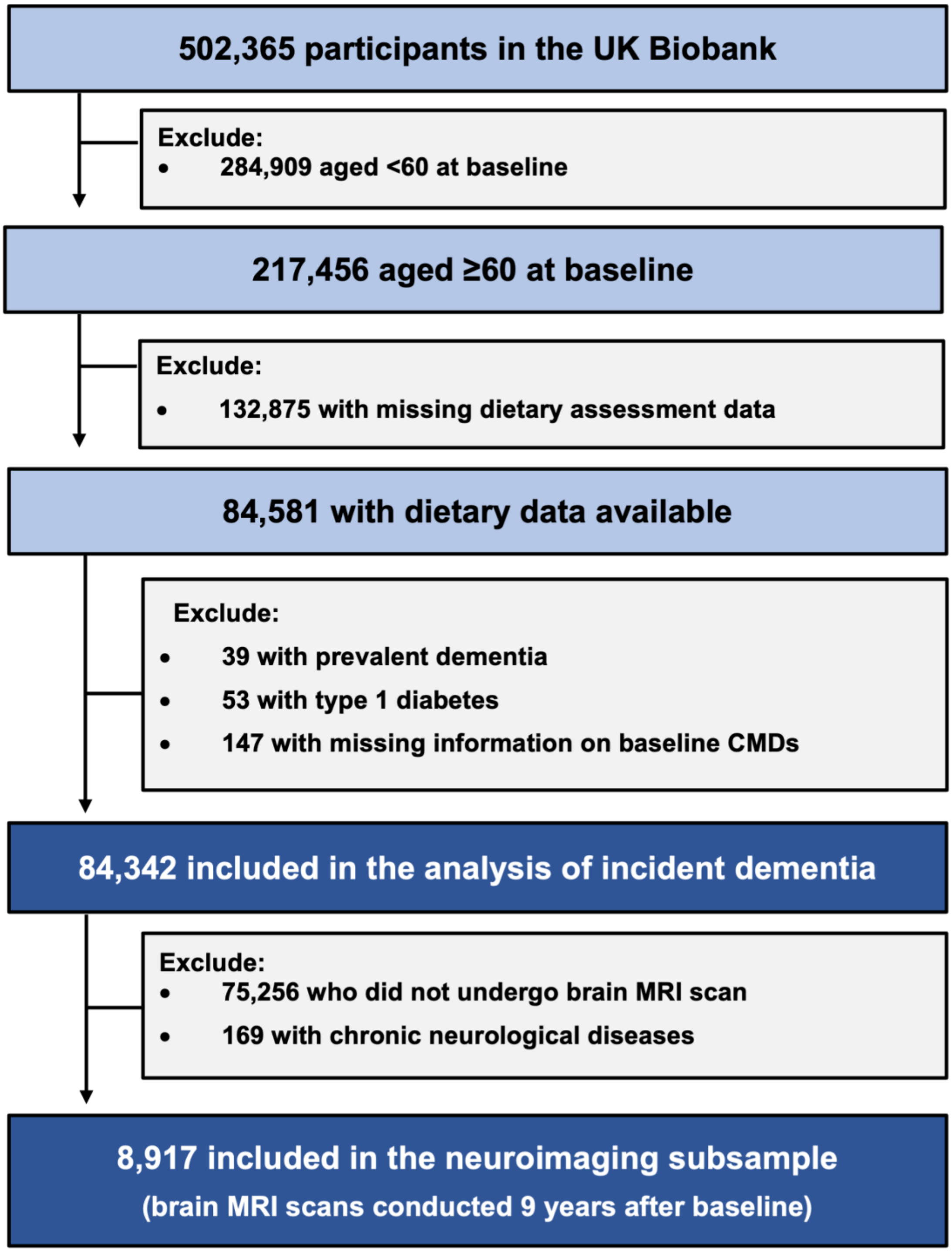

© 2024 Dove A et al. *JAMA Network Open.*

eTable 1. Neurological disorders used as exclusion criteria for the neuroimaging subsample.

Neurological disorder (self-reported)	Code (Field ID 20002 and 20003)
Parkinson's disease	1262
Dementia or Alzheimer's disease	1263
Chronic degenerative neurological	1258
Guillain-Barré syndrome	1256
Multiple Sclerosis	1261
Other demyelinating disease	1397
Brain cancer	1032
Brain/intracranial abscess	1245
Cerebral aneurysm	1425
Cerebral palsy	1433
Encephalitis	1246
Epilepsy	1264
Head injury	1266
Infections of the nervous system	1244
Meningeal cancer	1031
Meningioma (benign)	1659
Meningitis	1247
Motor Neuron Disease	1259
Neurological injury/trauma	1240
Spina bifida	1524
Subdural hematoma	1083

© 2024 Dove A et al. *JAMA Network Open.*

eTable 2. UK Biobank Field Codes used to ascertain baseline CMD status.

	Medical Records (ICD-10 codes)	Self-Reported Medical History	Self-Reported Medications	Biochemical Measures
**Type 2 diabetes**	130709 (E11)<br>130711 (E12)<br>130713 (E13)<br>130715 (E14)	2443<br>2976	6153<br>6177	30750 (HbA1c ≥6.5%)
**Heart disease**	131297 (I20)<br>131299 (I21)<br>131301 (I22)<br>131303 (I23)<br>131305 (I24)<br>131307 (I25)<br>131351 (I48)<br>131353 (I49)<br>131355 (I50)	3627<br>3894<br>6150		
**Stroke**	131361 (I60)<br>131363 (I61)<br>131365 (I62)<br>131367 (I63)<br>131375 (I67)<br>131367 (I68)	4056		

© 2024 Dove A et al. *JAMA Network Open.*

eTable 3. Inflammatory effect scores and globally daily mean intake for 45 dietary parameters included in the Dietary Inflammatory Index (DII).

Dietary parameter	Inflammatory effect score	Global daily mean intake (units/day)
Alcohol (g)	-0.278	13.98 ± 3.72
Anthocyanidins (mg)	-0.131	18.05 ± 21.14
β-carotene (μg)	-0.584	3718 ± 1720
Caffeine (g)	-0.110	8.05 ± 6.67
Carbohydrate (g)	0.097	272.2 ± 40.0
Cholesterol (mg)	0.110	279.4 ± 51.2
Energy (kcal)	0.180	2056 ± 338
Eugenol (mg)	-0.140	0.01 ± 0.08
Total fat (g)	0.298	71.4 ± 19.4
Fiber (g)	-0.663	18.8 ± 4.9
Flavan-3-ol (mg)	-0.415	95.8 ± 85.9
Flavones (mg)	-0.616	1.55 ± 0.07
Flavonols (mg)	-0.467	17.70 ± 6.79
Flavonones (mg)	-0.250	11.70 ± 3.82
Folic acid (μg)	-0.190	273.0 ± 70.7
Garlic (g)	-0.412	4.35 ± 2.90
Ginger (g)	-0.453	59.0 ± 63.2
Green/black tea (g)	-0.536	1.69 ± 1.53
Iron (mg)	0.032	13.35 ± 3.71
Isoflavones (mg)	-0.593	1.20 ± 0.20
Magnesium (mg)	-0.484	310.1 ± 139.4
Monounsaturated fatty acids (g)	-0.009	27.0 ± 6.1
Niacin (mg)	-0.246	25.90 ± 11.77
Omega-3 fatty acids (g)	-0.436	1.06 ± 1.06
Omega-6 fatty acids (g)	-0.159	10.80 ± 7.50
Onion (g)	-0.301	35.9 ± 18.4
Pepper (g)	-0.131	10.0 ± 7.07
Polyunsaturated fatty acids (g)	-0.337	13.88 ± 3.76
Protein (g)	0.021	79.4 ± 13.9
Riboflavin (mg)	-0.068	1.70 ± 0.79
Rosemary (mg)	-0.013	1.00 ± 15.00
Saffron (g)	-0.140	0.37 ± 1.78
Saturated fat (g)	0.373	28.6 ± 8.0
Selenium (μg)	-0.191	67.0 ± 25.1
Thiamin (mg)	-0.098	1.70 ± 0.66
Thyme/oregano (mg)	-0.102	0.33 ± 0.99
Trans fat (g)	0.229	3.15 ± 3.75
Turmeric (mg)	-0.785	533.6 ± 754.3
Vitamin A (RE)	-0.401	983.9 ± 518.6
Vitamin B6 (mg)	-0.365	1.47 ± 0.74
Vitamin B12 (μg)	0.106	5.15 ± 2.70
Vitamin C (mg)	-0.424	118.2 ± 43.46
Vitamin D (μg)	-0.446	6.26 ± 2.21
Vitamin E (mg)	-0.419	8.73 ± 1.49
Zinc (mg)	-0.313	9.84 ± 2.19

Table modified from Shivappa *et al*. 2014 (doi:10.1017/S1368980013002115). Negative values indicate an anti-inflammatory effect and positive values indicate a pro-inflammatory effect. Shaded boxes indicate items for which data is available in the UK Biobank.

Abbreviations: g=grams; mg=milligrams; RE=retinol equivalents; μg=micrograms.

© 2024 Dove A et al. *JAMA Network Open.*

eTable 4. UK Biobank brain MRI image acquisition protocols.

Modality	Duration	Voxel, Matrix	Key Parameters
T1	5 minutes	1×1×1 mm<br>208×256×256	3D MPRAGE, sagittal, R=2, TI/TR=880/2000 ms
T2 FLAIR	6 minutes	1.05×1.0×1.0 mm<br>192×256×256	FLAIR, 3D SPACE, sagittal, R=2, PF 7/8, fat sat, TI/TR=1800/5000 ms, elliptical

All non-EPI scans are pre-scan normalized (on-scanner bias-field corrected). Gradient distortion correction is deselected on the scanner and applied in post-processing.

© 2024 Dove A et al. *JAMA Network Open.*

eTable 5. Baseline characteristics of the neuroimaging subsample (n=8,917).

Characteristics	Overall (n=8,917)	By CMD status		
		CMD-free (n=7,773)	CMDs (n=1,144)	P-value
Age, years	63.4 ± 2.7	63.3 ± 2.7	63.9 ± 2.8	0.419
Sex				<0.001
Female	4,078 (45.7)	3,744 (48.2)	334 (29.2)	
Male	4,839 (54.3)	4,029 (51.8)	810 (70.8)	
College/university-educated	4,089 (46.0)	3,588 (46.3)	501 (44.0)	0.142
White race/ethnicity	8,401 (94.5)	7,333 (94.6)	1,068 (93.7)	0.184
Townsend deprivation index	-2.2 ± 2.6	-2.2 ± 2.5	-1.9 ± 4.3	0.001
Body mass index (BMI), kg/m^2	26.5 ± 3.9	26.3 ± 3.7	28.3 ± 4.9	<0.001
Underweight (<20)	183 (2.1)	179 (2.3)	4 (0.4)	<0.001
Normal (20 – 25)	3,174 (35.6)	2,919 (37.6)	255 (22.3)	
Overweight (25 – 30)	4,103 (46.1)	3,554 (45.8)	549 (48.0)	
Obese (≥30)	1,448 (16.3)	1,112 (14.3)	336 (29.4)	
Smoking				<0.001
Never	4,902 (55.1)	4,375 (56.4)	527 (46.2)	
Former	3,614 (40.6)	2,886 (43.0)	411 (41.9)	
Current	380 (4.3)	2,809 (41.9)	382 (38.9)	
Physical activity				0.003
Low	1,203 (15.6)	1,104 (15.1)	189 (19.3)	
Moderate	3,297 (42.9)	25,249 (43.3)	4,986 (42.7)	
High	3,191 (41.5)	23,883 (40.9)	4,197 (36.0)	
Hypertension	2,713 (30.5)	2,068 (26.6)	645 (56.7)	<0.001
APOE ε4 carrier	1,950 (26.0)	1,696 (25.9)	254 (26.7)	0.599
Energy intake, kcal	2,069 ± 495	2,067 ± 493	2,086 ± 502	0.231
Dietary inflammatory index (DII)	-0.8 ± 1.9	-0.8 ± 1.9	-0.8 ± 1.8	0.7583
Pro-inflammatory (DII ≥0.5 pts)	3,367 (37.8)	1,959 (25.2)	276 (24.1)	0.216
Neutral (DII >-1.5 to <0.5 pts)	3,315 (37.2)	2,863 (36.8)	452 (39.5)	
Anti-inflammatory (DII ≤-1.5 pts)	2,235 (25.1)	2,951 (38.0)	416 (36.4)	

Data are presented as means ± standard deviations or number (proportion, %).

Missing data: 30 for education level; 29 for race/ethnicity; 4 for Townsend deprivation index; 9 for BMI; 21 for smoking status; 1,226 for physical activity level; 6 for hypertension; 1,404 for APOE ε4 status

© 2024 Dove A et al. *JAMA Network Open.*

eTable 6. Comparison of participants included vs. not included in the neuroimaging subsample.

Characteristics	**Not Included in Neuroimaging Subsample** (n=75,425)	**Included in Neuroimaging Subsample** (n=8,917)	P-value
Age, years	63.9 ± 2.8	63.4 ± 2.7	<0.001
Sex			<0.001
Female	39,142 (51.9)	4,078 (45.7)	
Male	36,283 (48.1)	4,839 (54.3)	
College/university-educated	26,787 (35.7)	4,089 (46.0)	<0.001
White race/ethnicity	70,186 (93.4)	8,401 (94.5)	<0.001
Townsend deprivation index	-1.8 ± 2.7	-2.2 ± 2.6	<0.001
Body mass index (BMI), kg/m^2	27.2 ± 4.4	26.5 ± 3.9	<0.001
Underweight (<20)	1,502 (2.0)	182 (2.1)	<0.001
Normal (20 – 25)	23,583 (31.4)	3,174 (35.6)	
Overweight (25 – 30)	33,531 (44.6)	4,103 (46.1)	
Obese (≥30)	16,579 (22.1)	1,448 (16.3)	
Smoking			<0.001
Never	38,255 (50.9)	4,902 (55.1)	
Former	32,370 (43.0)	3,614 (40.6)	
Current	4,580 (6.1)	380 (4.3)	
Physical activity			0.006
Low	10,495 (16.8)	1,203 (15.6)	
Moderate	26,938 (43.2)	3,297 (42.9)	
High	24,889 (39.9)	3,191 (41.5)	
CMDs	12,935 (17.2)	1,144 (12.8)	<0.001
Hypertension	26,898 (35.7)	2,713 (30.5)	<0.001
APOE ε4 carrier	17,366 (27.9)	1,950 (26.0)	<0.001
Energy intake, kcal	2,031 ± 521	2,069 ± 495	<0.001
Dietary inflammatory index (DII)	-0.6 ± 2.0	-0.8 ± 1.9	<0.001
Pro-inflammatory (DII ≥0.5 pts)	22,182 (29.4)	2,235 (25.1)	<0.001
Neutral (DII >-1.5 to <0.5 pts)	26,709 (35.4)	3,315 (37.2)	
Anti-inflammatory (DII ≤-1.5 pts)	26,534 (35.2)	3,367 (37.8)	

Data are presented as means ± standard deviations or number (proportion, %).

Missing data: 515 for education level; 330 for race/ethnicity; 76 for Townsend deprivation index; 239 for BMI; 241 for smoking status; 14,329 for physical activity level; 121 for hypertension; 14,566 for APOE ε4 status

© 2024 Dove A et al. *JAMA Network Open.*

eTable 7. Summary of main results using non-imputed data for covariates.

Joint Exposure		Dementia		Brain MRI Measures					
				n	Total Brain Volume	Gray Matter Volume	White Matter Volume	Hippocampal Volume	WMH Volume
CMDs	*Dietary inflammatory potential*	*n*	HR (95% CI)		β (95% CI)	β (95% CI)	β (95% CI)	β (95% CI)	β (95% CI)
CMD-free	Anti-inflammatory	24,941	Reference	2,951	Reference	Reference	Reference	Reference	Reference
CMD-free	Neutral	25,200	1.02 (0.86, 1.23)	2,863	0.00 (-0.05, 0.05)	0.01 (-0.05, 0.06)	-0.00 (-0.05, 0.05)	0.02 (-0.04, 0.08)	0.01 (-0.04, 0.07)
CMD-free	Pro-inflammatory	20,122	1.20 (0.98, 1.47)	1,959	-0.03 (-0.09, 0.03)	-0.02 (-0.09, 0.04)	-0.03 (-0.10, 0.03)	-0.04 (-0.11, 0.03)	0.06 (-0.01, 0.13)
CMDs	Anti-inflammatory	4,960	**1.63 (1.29, 2.06)**	416	-0.09 (-0.18, 0.01)	**-0.13 (-0.23, -0.02)**	-0.04 (-0.14, 0.06)	-0.12 (-0.23, 0.01)	0.06 (-0.05, 0.17)
CMDs	Neutral	4,824	**1.95 (1.53, 2.48)**	452	**-0.19 (-0.28, -0.09)**	**-0.21 (-0.31, -0.11)**	**-0.14 (-0.24, -0.05)**	**-0.16 (-0.27, -0.04)**	0.05 (-0.05, 0.16)
CMDs	Pro-inflammatory	4,295	**2.39 (1.83, 3.11)**	276	**-0.17 (-0.30, -0.05)**	**-0.23 (-0.36, -0.10)**	-0.11 (-0.23, 0.02)	-0.02 (-0.17, 0.13)	**0.15 (0.02, 0.28)**

All models were adjusted for age at baseline, sex, education, energy intake, race/ethnicity, socioeconomic status, body mass index, smoking status, physical activity, hypertension, and *APOE* ε4 carrier status. Brain MRI analyses were additionally adjusted for head position, scanner table position, and assessment center.

© 2024 Dove A et al. *JAMA Network Open.*

eTable 8. Hazard ratios (HR) from Cox regression models for the associations of CMD status and dietary inflammatory potential with dementia risk, after excluding 109 study participants who developed dementia within the first 5 years of follow-up (n=84,233).

		No. of subjects	HR of all-cause dementia	
			Basic Adjusted	Multi-Adjusted
**CMD status**				
CMD-free		70,195	Reference	Reference
1 CMD		12,205	**1.64 (1.45 – 1.86)**	**1.61 (1.41 – 1.83)**
≥2 CMDs		1,833	**2.84 (2.28 – 3.54)**	**2.69 (2.14 – 3.38)**
**Dietary inflammatory potential**				
Pro-inflammatory (DII ≥0.5 pts)		24,374	Reference	Reference
Neutral (DII >-1.5 to <0.5 pts)		29,991	**0.76 (0.75 – 0.98)**	**0.87 (0.76 – 9.99)**
Anti-inflammatory (DII ≤-1.5 pts)		29,868	**0.83 (0.71 – 0.96)**	**0.82 (0.70 – 0.95)**
**Joint Exposure**				
CMDs	*Dietary inflammatory potential*			
CMD-free	Anti-inflammatory	24,916	Reference	Reference
CMD-free	Neutral	25,180	1.03 (0.88 – 1.19)	1.04 (0.90 – 1.21)
CMD-free	Pro-inflammatory	20,099	1.18 (0.99 – 1.40)	1.19 (0.99 – 1.41)
CMDs	Anti-inflammatory	4,952	**1.69 (1.39 – 2.06)**	**1.64 (1.35 – 2.00)**
CMDs	Neutral	4,811	**1.90 (1.55 – 2.32)**	**1.83 (1.50 – 2.25)**
CMDs	Pro-inflammatory	4,275	**2.21 (1.78 – 2.74)**	**2.16 (1.73 – 2.69)**

Basic-adjusted models included age at baseline, sex, education, and energy intake. Multi-adjusted models additionally included race/ethnicity, socioeconomic status, body mass index, smoking status, physical activity, hypertension, *APOE* ε4 carrier status, and CMD status or DII, as appropriate.

© 2024 Dove A et al. *JAMA Network Open.*

eTable 9. Subdistribution hazard ratios (SHR) from Fine & Gray regression models for the associations of CMD status and dietary inflammatory potential with dementia risk, accounting for the competing risk of death.

		No. of subjects	HR of all-cause dementia	
			Basic Adjusted	Multi-Adjusted
**CMD status**				
CMD-free		70,263	Reference	Reference
1 CMD		12,236	**1.65 (1.47 – 1.87)**	**1.64 (1.45 – 1.85)**
≥2 CMDs		1,843	**2.74 (2.22 – 3.38)**	**2.65 (2.13 – 3.31)**
**Dietary inflammatory potential**				
Pro-inflammatory (DII ≥0.5 pts)		24,417	Reference	Reference
Neutral (DII >-1.5 to <0.5 pts)		30,024	**0.85 (0.75 – 0.97)**	**0.85 (0.75 – 0.97)**
Anti-inflammatory (DII ≤-1.5 pts)		29,901	**0.82 (0.71 – 0.94)**	**0.80 (0.69 – 0.93)**
**Joint Exposure**				
CMDs	*Dietary inflammatory potential*			
CMD-free	Anti-inflammatory	24,941	Reference	Reference
CMD-free	Neutral	25,200	1.01 (0.87 – 1.17)	1.03 (0.89 – 1.19)
CMD-free	Pro-inflammatory	20,122	1.17 (0.99 – 1.38)	**1.18 (1.01 – 1.40)**
CMDs	Anti-inflammatory	4,960	**1.63 (1.35 – 1.97)**	**1.60 (1.32 – 1.94)**
CMDs	Neutral	4,824	**1.89 (1.55 – 2.29)**	**1.86 (1.53 – 2.26)**
CMDs	Pro-inflammatory	4,295	**2.26 (1.84 – 2.77)**	**2.25 (1.83 – 2.78)**

Basic-adjusted models included age at baseline, sex, education, and energy intake. Multi-adjusted models additionally included race/ethnicity, socioeconomic status, body mass index, smoking status, physical activity, hypertension, *APOE* ε4 carrier status, and CMD status or DII, as appropriate.

eTable 10. Association between dietary inflammatory potential and CRP: results from linear regression models.

**Dietary inflammatory potential**	**No. of participants**	**Unadjusted** β (95% CI)	**Basic-Adjusted** β (95% CI)	**Multi-Adjusted** β (95% CI)
Anti-inflammatory (DII ≤-1.5 pts)	29,901	Reference	Reference	Reference
Neutral (DII >-1.5 to <0.5 pts)	30,024	**0.13 (0.05, 0.20)**	**0.15 (0.08, 0.23)**	**0.08 (0.01, 0.15)**
Pro-inflammatory (DII ≥0.5 pts)	24,417	**0.46 (0.39, 0.54)**	**0.53 (0.44, 0.61)**	**0.32 (0.23, 0.41)**
Higher DII score		**0.10 (0.09, 0.12)**	**0.13 (0.11, 0.15)**	**0.08 (0.06, 0.10)**

Basic-adjusted models included age at baseline, sex, education, and energy intake. Multi-adjusted models additionally included race/ethnicity, socioeconomic status, body mass index, smoking status, physical activity, hypertension, and *APOE* ε4 carrier status.

© 2024 Dove A et al. *JAMA Network Open.*

eTable 11. DII scores over multiple assessments in the anti-inflammatory, neutral, and pro-inflammatory diet groups.

		DII_0	DII_1	DII_2	DII_3	DII_4
**Anti-inflammatory**	*n*	10,182	14,415	11,891	15,059	14,629
	Mean	-2.71 ± 1.22	-2.42 ± 1.89	-2.43 ± 1.39	-2.39 ± 1.39	-2.40 ± 1.4
**Neutral**	*n*	9,983	14,961	12,706	15,591	15,199
	Mean	-0.79 ± 1.06	-0.58 ± 1.26	-0.61 ± 1.29	-0.58 ± 1.28	-0.60 ± 1.26
**Pro-inflammatory**	*n*	9,010	10,211	8,479	10,352	10,101
	Mean	1.19 ± 1.06	1.17 ± 1.25	1.13 ± 1.27	1.15 ± 1.26	1.17 ± 1.25
**Total**	*n*	29,175	39,587	33,076	41,002	39,929
	Mean	-0.85 ± 1.95	-0.80 ± 1.92	-0.82 ± 1.92	-0.81 ± 1.91	-0.81 ± 1.91

© 2024 Dove A et al. *JAMA Network Open.*

eFigure 2. Trajectories of DII scores over multiple assessments in the anti-inflammatory, neutral, and pro-inflammatory diet groups.

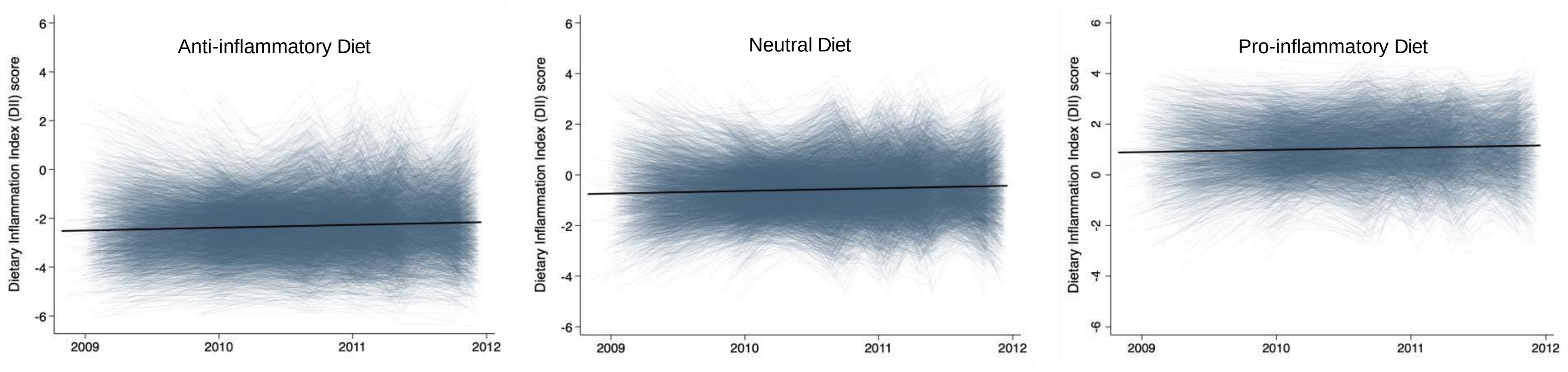

Spaghetti plots include data from participants in the anti-inflammatory (DII_{avg} ≤-1.5 pts), neutral (DII_{avg} >-1.5 to <0.5 pts), and pro-inflammatory (DII_{avg} ≥0.5 pts) diet groups for whom DII information was available baseline and at least one additional assessment. Each thin blue line represents the trajectory of one participant's DII scores over multiple assessments. The thick black lines represent the trend in DII score over time.

© 2024 Dove A et al. *JAMA Network Open.*

eTable 12. Summary of main results including only participants who completed two or more dietary assessments (n=51,182).

**Joint Exposure**		**Dementia**			**Brain MRI Measures**				
					**Total Brain Volume**	**Gray Matter Volume**	**White Matter Volume**	**Hippocampal Volume**	**WMH Volume**
CMDs	*Dietary inflammatory potential*	*n*	HR (95% CI)	*n*	β (95% CI)	β (95% CI)	β (95% CI)	β (95% CI)	β (95% CI)
CMD-free	Anti-inflammatory	16,005	Reference	2,222	Reference	Reference	Reference	Reference	Reference
CMD-free	Neutral	16,539	1.06 (0.87, 1.30)	2,223	-0.02 (-0.07, 0.03)	-0.03 (-0.08, 0.02)	-0.01 (-0.05, 0.04)	-0.02 (-0.08, 0.04)	0.01 (-0.05, 0.06)
CMD-free	Pro-inflammatory	10,678	**1.35 (1.07, 1.70)**	1,313	-0.03 (-0.09, 0.03)	-0.04 (-0.10, 0.03)	-0.02 (-0.08, 0.04)	-0.05 (-0.13, 0.02)	0.04 (-0.02, 0.11)
CMDs	Anti-inflammatory	2,966	**1.64 (1.24, 2.15)**	316	**-0.10 (-0.19, -0.01)**	**-0.13 (-0.23, -0.03)**	-0.06 (-0.16, 0.03)	**-0.17 (-0.28, 0-.06)**	0.02 (-0.08, 0.13)
CMDs	Neutral	2,958	**2.25 (1.73, 2.93)**	348	**-0.14 (-0.23, -0.05)**	**-0.18 (-0.28, -0.08)**	-0.09 (-0.18, 0.01)	**-0.13 (-0.24, -0.02)**	0.06 (-0.04, 0.16)
CMDs	Pro-inflammatory	2,036	**3.01 (2.23, 4.07)**	179	**-0.18 (-0.31, -0.05)**	**-0.26 (-0.39, -0.12)**	-0.10 (-0.22, 0.03)	**-0.16 (-0.31, -0.01)**	**0.21 (0.07, 0.35)**

All models were adjusted for age at baseline, sex, education, energy intake, race/ethnicity, socioeconomic status, body mass index, smoking status, physical activity, hypertension, and *APOE* ε4 carrier status. Brain MRI analyses were additionally adjusted for head position, scanner table position, and assessment center.

© 2024 Dove A et al. *JAMA Network Open.*

eTable 13. Summary of main results using DII score calculated based on only baseline dietary assessment (n=29,175).

**Joint Exposure**			**Dementia**	**Brain MRI Measures**					
				n	**Total Brain Volume**	**Gray Matter Volume**	**White Matter Volume**	**Hippocampal Volume**	**WMH Volume**
CMDs	*Dietary inflammatory potential*	*n*	HR (95% CI)		β (95% CI)	β (95% CI)	β (95% CI)	β (95% CI)	β (95% CI)
CMD-free	Anti-inflammatory	9,338	Reference	837	Reference	Reference	Reference	Reference	Reference
CMD-free	Neutral	8,554	1.18 (0.91, 1.53)	677	-0.01 (-0.09, 0.08)	0.01 (-0.08, 0.10)	-0.01 (-0.10, 0.07)	-0.09 (-0.19, 0.01)	-0.05 (-0.14, 0.04)
CMD-free	Pro-inflammatory	6,174	1.22 (0.90, 1.65)	414	-0.09 (-0.19, 0.01)	-0.10 (-0.21, 0.01)	-0.07 (-0.17, 0.03)	**-0.17 (-0.29, -0.05)**	-0.01 (-0.12, 0.10)
CMDs	Anti-inflammatory	2,025	**1.55 (1.10, 2.18)**	118	**-0.19 (-0.36, -0.03)**	**-0.29 (-0.46, -0.11)**	-0.09 (-0.26, 0.08)	-0.16 (-0.36, 0.04)	-0.09 (-0.27, 0.09)
CMDs	Neutral	1,687	**2.45 (1.77, 3.39)**	106	**-0.24 (-0.40, -0.08)**	**-0.24 (-0.40, -0.07)**	**-0.22 (-0.38, -0.06)**	-0.11 (-0.30, 0.07)	**-0.20 (-0.36, -0.03)**
CMDs	Pro-inflammatory	1,397	**2.81 (1.95, 4.04)**	54	**-0.27 (-0.50, -0.04)**	**-0.33 (-0.57, -0.09)**	-0.19 (-0.43, 0.04)	-0.02 (-0.29, 0.25)	0.15 (-0.10, 0.39)

All models were adjusted for age at baseline, sex, education, energy intake, race/ethnicity, socioeconomic status, body mass index, smoking status, physical activity, hypertension, and *APOE* ε4 carrier status. Brain MRI analyses were additionally adjusted for head position, scanner table position, and assessment center.

© 2024 Dove A et al. *JAMA Network Open.*
