# Supplementary material for: Anti-Inflammatory Diet and Dementia in Older Adults With Cardiometabolic Diseases
Source: JAMA Netw Open. 2024 Aug 12;7(8):e2427125. doi: 10.1001/jamanetworkopen.2024.27125 (PMC11320167; doi:10.1001/jamanetworkopen.2024.27125)
Supplement: Supplement 2. — Data Sharing Statement [file jamanetwopen-e2427125-s002.pdf]

Data Sharing Statement

Dove. Anti-Inflammatory Diet and Dementia in Older Adults With Cardiometabolic Diseases. *JAMA Netw Open*. Published August 12, 2024. doi:10.1001/jamanetworkopen.2024.27125

Data
Data available: No

Additional Information
Explanation for why data not available: Requests for access to the UK Biobank data can be made here: https://www.ukbiobank.ac.uk/enable-your-research/apply-for-access.
